# Temporal Shifts in Hormone Signaling Networks Orchestrate Soybean Floral Development Under Field Conditions: An RNA-Seq Study

**DOI:** 10.3390/ijms26136455

**Published:** 2025-07-04

**Authors:** Eszter Virág, Géza Hegedűs, Ágnes Nagy, József Péter Pallos, Barbara Kutasy

**Affiliations:** 1 One Health Institute, Faculty of Health Science, University of Debrecen, Egyetem Sq. 1, 4032 Debrecen, Hungary; 2 Research Institute for Medicinal Plants and Herbs Ltd., Lupaszigeti Str. 4, 2011 Budakalász, Hungary; agnes.nagy5@pte.hu (Á.N.); pallos.jp@gynki.hu (J.P.P.); kutasy.barbara.julia@uni-mate.hu (B.K.); 3 Department of Information Technology and Its Applications, Faculty of Information Technology, University of Pannonia, 8900 Zalaegerszeg, Hungary; hegedus.geza@zek.uni-pannon.hu; 4 Department of Plant Physiology and Plant Ecology, Georgikon Campus, Institute of Agronomy, Hungarian University of Agriculture and Life Sciences, Festetics Str. 7, 8360 Keszthely, Hungary

**Keywords:** soybean breading, *Glycine max*, reproduction, MADS-Box, phytohormones, *GmNMH7*, flower development, transcriptome assembly, differential gene expression

## Abstract

Floral ontogeny in soybean (*Glycine max*) is governed by multilayered regulatory hierarchies that integrate phytohormonal cues with precisely choreographed gene-expression programs. Yet, the transcriptomic architecture underpinning this continuum remains only partially resolved. Here, we generated a strand-specific, high-depth temporal transcriptome atlas of soybean inflorescences spanning four morphologically defined stadiums (Stadium 0–Stadium 3). We detected transcriptional activity for 60,889 loci; pairwise stadium contrasts revealed 4000–7000 differentially expressed genes, with the most extensive reprogramming coinciding with the onset of anthesis (Stadium 2). Unsupervised clustering delineated ~600 genes peaking at the pre-anthesis phase (Stadium 1), a cohort enriched for transcriptional regulators and floral organ-identity determinants. Stadium-resolved gene-set enrichment and KEGG mapping uncovered dynamic modulation of canonical hormone-signaling pathways—including auxin, cytokinin, gibberellin, abscisic acid, ethylene, jasmonate, and salicylate circuits—reflecting shifting developmental priorities. Forty-five MADS-box transcription factor genes were expressed; notably, *JOINTLESS* was strongly induced at anthesis, while the root-predominant factor *GmNMH7* exhibited unexpected floral expression, implicating a hitherto unappreciated role in reproductive development. Quantitative RT-PCR of representative loci corroborated RNA-seq measurements. This high-resolution atlas refines our understanding of the hormonal and genetic circuitry of soybean floral morphogenesis, furnishing molecular targets for engineering flowering time and inflorescence architecture under fluctuating environmental conditions.

## 1. Introduction

The ontogeny of flowering in soybean (*Glycine max*) is a pivotal agronomic trait because it modulates multiple yield-determining parameters, from pod set to seed composition. Precise onset and duration of the floral phase constitute the most sensitive window of reproductive development; deviations from the optimal temporal or environmental framework can markedly diminish crop performance. Consequently, synchronizing floral initiation with favorable photothermal conditions and ensuring a sufficiently long anthesis period for effective pollination are prerequisites for maximizing yield and grain quality [1].

Phytohormone-signaling cascades are finely tuned by ambient photothermal cues [2]. Photoperiod exerts the dominant influence: soybean is a quantitative short-day species that initiates flowering once the nightly dark interval surpasses its critical threshold, thereby shifting hormonal equilibria toward floral induction. These photoperiodic cues recalibrate the endogenous hormone networks, thereby activating the genetic program that commits the shoot apex to flowering [3,4,5].

The transition from vegetative to reproductive growth in plants is tightly regulated by phytohormone biosynthesis and signaling pathways, primarily involving auxin (AUX), gibberellins (GA), cytokinins (CK), ethylene (ET), and abscisic acid (ABA) [6,7]. Auxins serve as key regulators of plant development, orchestrating cell elongation, division, and the maintenance of tissue polarity and symmetry [8,9,10]. In soybean, auxin plays a complex role in floral induction, functioning in synergy with cytokinins to promote flower formation and regulate the initiation of floral meristems and bud development [5,11]. However, AUX levels and activity are sensitive to environmental conditions, and various abiotic stresses can perturb AUX dynamics at distinct stages of floral development, potentially impacting reproductive outcomes [12]. Gas play a pivotal role in promoting the floral transition by activating the expression of florigen-associated genes, which constitute a central component of the flowering signal cascade [13]. In addition to their role in floral induction, Gas stimulate stem elongation and contribute to the development and maturation of floral organs, including the promotion of flower opening. Experimental application of GA biosynthesis inhibitors, such as paclobutrazol, has been shown to delay flowering in soybean, further substantiating the involvement of Gas in the regulation of floral initiation and developmental timing [14,15]. CKs exert a more indirect influence on flowering compared to Gas and AUXs. They are essential regulators of cell division and growth, particularly in promoting lateral bud outgrowth and meristem activity [16]. Emerging evidence suggests that CKs may also contribute to the vegetative-to-reproductive transition in plants, including soybean [17,18]. However, elevated CK levels can, in some cases, delay or reduce flowering by reinforcing apical dominance and suppressing floral meristem initiation [19]. CK signaling is intricately interconnected with both GA and AUX pathways, underscoring the complexity of hormonal crosstalk in the fine-tuning of flowering time and floral development [19,20,21]. ABA functions as a central stress-responsive hormone, orchestrating molecular and physiological adaptations to environmental challenges such as elevated temperatures and water deficits [22,23]. ABA levels often rise prior to flowering, potentially facilitating acclimation to suboptimal conditions. While ABA is not a primary regulator of floral induction, its role in modulating stress tolerance can indirectly influence flowering outcomes [24,25,26,27]. Interactions between ABA and other phytohormones, particularly Gas, remain an active area of investigation [21,28]. Although the mechanistic basis is not fully resolved, current evidence suggests that ABA and GA may function antagonistically in the regulation of flowering time, with ABA generally acting to suppress, and GA to promote, floral transition under stress conditions [29,30]. ET, traditionally associated with fruit ripening and senescence, has also been implicated in the regulation of flowering, although its precise role in soybean remains incompletely understood. Emerging studies indicate that ET may influence the timing of floral induction under specific environmental stress conditions, such as elevated humidity or temperature fluctuations [31,32,33]. These findings suggest a potential modulatory function for ET in stress-responsive flowering pathways, warranting further investigation into its context-dependent regulatory mechanisms. JA is a key phytohormone involved in numerous aspects of plant development, including the regulation of floral development across diverse species, including soybean [34]. In soybean, the role of JA in flowering is complex and not yet fully elucidated. Emerging evidence suggests that JA can delay the onset of flowering, particularly when applied exogenously, likely due to its involvement in modulating cell growth, differentiation, and stress responses—factors that may postpone the vegetative-to-reproductive phase transition [35,36,37,38,39].

Despite this potential delay in floral initiation, JA also plays a crucial role during the later stages of reproductive development, particularly in the differentiation and maturation of floral organs such as petals, stamens, and pistils [40]. Studies in model systems have demonstrated that JA influences floral organ identity by regulating the expression of key transcription factors, suggesting a conserved developmental role [41,42]. In soybean, JA has additionally been linked to flower and pod abscission, especially under environmental stress or resource-limited conditions.

Overall, the effects of JA on soybean flowering appear to be highly context-dependent, with both inhibitory and promotive roles depending on developmental stage, hormone balance, and environmental conditions.

Ongoing research into JA and its crosstalk with other phytohormonal pathways is expected to yield a more nuanced understanding of its regulatory functions in soybean flowering. Elucidating these interactions will be crucial for defining the precise role of JA in coordinating floral development, particularly under abiotic stress conditions. Such insights may offer novel strategies for manipulating hormonal networks to optimize flowering time, enhance reproductive success, and improve overall crop resilience and productivity. The role of SA in soybean flowering remains comparatively underexplored relative to other phytohormones. Nonetheless, emerging evidence indicates that SA can influence floral development through multiple regulatory pathways. Several studies have shown that both exogenous application and elevated endogenous levels of SA may delay flowering in soybean and other species [43,44,45]. This delay is hypothesized to result, at least in part, from SA-mediated suppression of GA signaling, a pathway known to promote floral transition [46]. Under pathogen-induced stress, the accumulation of SA may prioritize defense responses over reproduction, thereby postponing flowering to enhance immune function [46].

In addition to its interaction with GA, SA has been reported to modulate CK signaling, potentially influencing the differentiation and morphogenesis of floral structures [47,48]. Furthermore, SA may affect AUX signaling pathways, which are critical for organ patterning and floral development [49]. These multifaceted interactions suggest that SA operates as a hormonal modulator capable of integrating environmental stress cues with developmental outcomes. Depending on environmental conditions and the physiological state of the plant, SA may either inhibit or support aspects of floral development [50,51,52]. However, the precise molecular mechanisms by which SA influences soybean flowering remain to be fully elucidated and warrant further investigation.

The genetic regulation of phytohormone signaling during soybean floral development is governed by a complex and tightly integrated network that coordinates the spatial and temporal progression of floral organogenesis. Core phytohormones—including Gas, AUXs, CKs, ABA, ET, SA, and JA—interact with key flowering-time regulators to mediate floral induction and organ differentiation in response to environmental stimuli such as photoperiod and abiotic stress. Deciphering the genetic architecture underlying these hormone-mediated interactions is crucial for developing breeding strategies that enhance flowering efficiency and stabilize yield under variable environmental conditions.

To this end, the present study employs high-resolution temporal RNA-Seq analysis across four distinct stages of floral development in *Glycine max*, with a specific emphasis on the expression dynamics of phytohormone-associated genes. The soybean cultivar ES Director was used in field cultivation representing an optimal choice for field-based research in Hungary due to its reliable agronomic performance and adaptability to Central European climatic conditions. As a mid-season cultivar developed for temperate regions, ES Director aligns well with Hungary’s growing season, ensuring consistent phenological development under local conditions. This stability is particularly valuable for transcriptomic, physiological, and agronomic studies where developmental timing and environmental responsiveness must be controlled.

We selected four morphologically defined floral developmental stages (Stadium 0 to Stadium 3) to capture the critical temporal transitions during soybean flower ontogeny under field conditions. These stages represent a continuum from floral meristem initiation (Stadium 0), through organ specification and early morphogenesis (Stadium 1), to peak anthesis (Stadium 2) and the onset of floral senescence (Stadium 3). By focusing on these well-separated stadiums, we aimed to elucidate the dynamic gene expression patterns and hormone signaling networks that orchestrate floral development, including both the inductive and terminal phases of reproductive differentiation. This temporal resolution enabled the identification of stage-specific regulatory genes and hormonal shifts, providing novel insights into the coordination of developmental and environmental cues in the regulation of flowering.

## 2. Results

### 2.1. Reference Guided Assembly of Transcripts and Re-Annotation of Mapped Genes

Transcriptomes from floral samples collected at four distinct developmental stages (stadium) under field conditions were analyzed using high-throughput RNA-Seq technology. For each developmental stage, 15–20 million single end 100-bp Illumina reads were generated from mRNA extracted from the corresponding floral samples.

**Figure 1:**
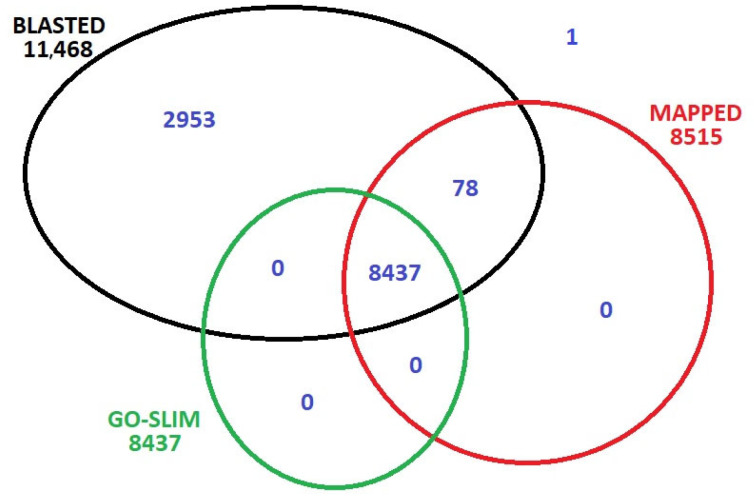
Data distribution after the re-annotation of mapped 60,889 genes to the reference genome.

Reads were screened to a quality score of Phred Q30  >  100% from the four stadiums, retaining 93% of sequencing reads. Cleaned reads were mapped to the reference genome GCF_000004515.6, downloaded from NCBI 11.11.2024, using Bowtie2. The average library size across the four analyzed floral samples was approximately 2,074,467 reads. Coding sequences (CDS) and reads that matched were retained for downstream analyses. The mapped genes were re-annotated using BLAST 2.16.0+ (BLASTX, Viridiplantae database), and Gene Ontology (GO). A total of 60,889 genes were identified and annotated across the four stages of floral development. Of these, 73% were successfully assigned to at least one Plant GO-Slim category, while 26% exhibited significant sequence similarity to known proteins based on BLAST searches. Only a single gene lacked a considerable BLAST hit (Figure 1).

### 2.2. Analysis of Transcript Abundance Across Four Developmental Stages of Soybean Flowers

**Figure 2:**
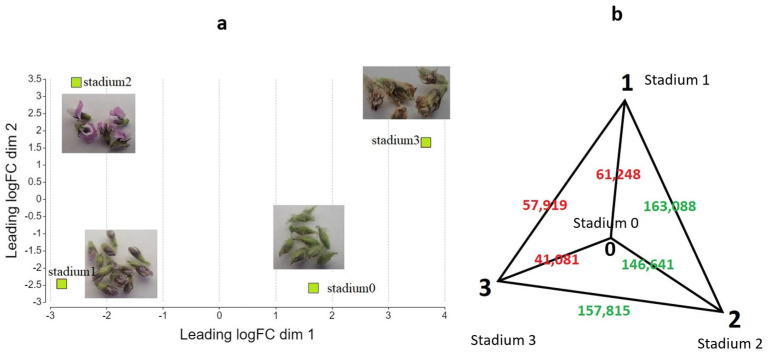
Two-dimensional scatter plot of sample distances resulting from a pairwise DEG analysis using a contrast of Stadium 1 versus Stadium 0 (**a**). Euclidean distance analysis of transcript abundance matrix (**b**). Stadium 2 exhibited the greatest divergence in expression profiles, showing approximately a threefold distance from the other samples. The values displayed in the figure represent the Euclidean distance of the abundance vectors belonging to each stage, with red indicating proximity and green indicating greater distance.

Genome-wide transcript abundance profiles were analyzed based on RNA-Seq data. We performed pairwise gene expression data analysis, investigating four sample pairs: Stadium 1 vs. Stadium 0, Stadium 2 vs. Stadium 0, Stadium 3 vs. Stadium 0, and Stadium 3 vs. Stadium 2. Distances of the typical log2 fold changes between samples are plotted in a two-dimensional scatterplot (Figure 2a). Euclidean norm (quadratic norm) analysis of the transcript abundance matrix revealed that Stadium 2 exhibited the most significant overall divergence in gene expression compared to the other developmental stadiums, with approximately a threefold increase in distance. The greatest difference in gene expression levels was observed between Stadium 1 and Stadium 2, as illustrated in Figure 2b. The number of total transcripts identified was 24852, and the number of expressed genes in each stadium was 13,408 (Stadium 0), 12,914 (Stadium 1), 12,829 (Stadium 2) and 10,743 (Stadium 3).

### 2.3. Overview of Differential Gene Expression (DEGs) Across Four Developmental Stadiums of Soybean Flowers

**Figure 3:**
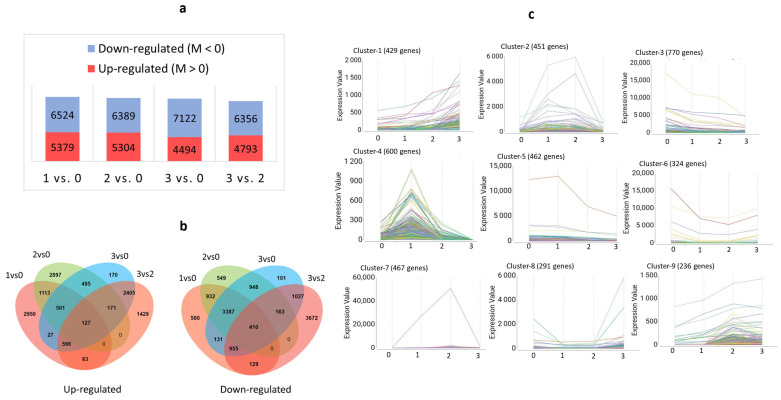
Pairwise expression analysis and time-course expression analysis of DEGs during soybean flower maturation. Diagram (**a**) shows the up- and downregulated DEG number changes across the four analyzed sample pairs. An M-value greater than 0, calculated as M = log_2_ (Expression in condition 1/Expression in condition 2), indicates that the gene is more highly expressed in condition 1 compared to condition 2 in the pairwise comparison. Venn diagram (**b**) illustrates the overlap and specificity of differentially upregulated and downregulated genes across the developmental stadium comparisons. Image (**c**) displays the clustered gene expression patterns. The clustered gene groups are represented in different colors in the pictures of Cluster 1–9.

We found 5379 up- and 6524 downregulated DEGs in the sample pair Stadium 1 vs. Stadium 0, 5304 up- and 6389 downregulated DEGs in Stadium 2 vs. Stadium 0, 4494 up- and 7122 downregulated DEGs in Stadium 3 vs. Stadium 0, and 4793 up- and 6356 downregulated DEGs in Stadium 3 vs. Stadium 2 (Figure 3a). Across the sample pairs, we found 124 upregulated and 410 downregulated common DEGs. Among the upregulated DEGs, the highest number of uniquely expressed genes was identified in the Stadium 1 vs. Stadium 0 comparison. In contrast, the lowest number was observed in the Stadium 3 vs. Stadium 0 comparison. Among the downregulated genes, the largest and smallest number of unique DEGs was found in Stadium 3 vs. Stadium 2 and Stadium 3 vs. Stadium 0, respectively (Figure 3b). The temporal expression patterns fell into nine major clusters. Among these, cluster 4—comprising 600 genes—exhibited a distinct peak in expression at Stadium 1 (Figure 3c).

### 2.4. Analysis of Characteristically Expressed Gene Families in Cluster 4

**Figure 4:**
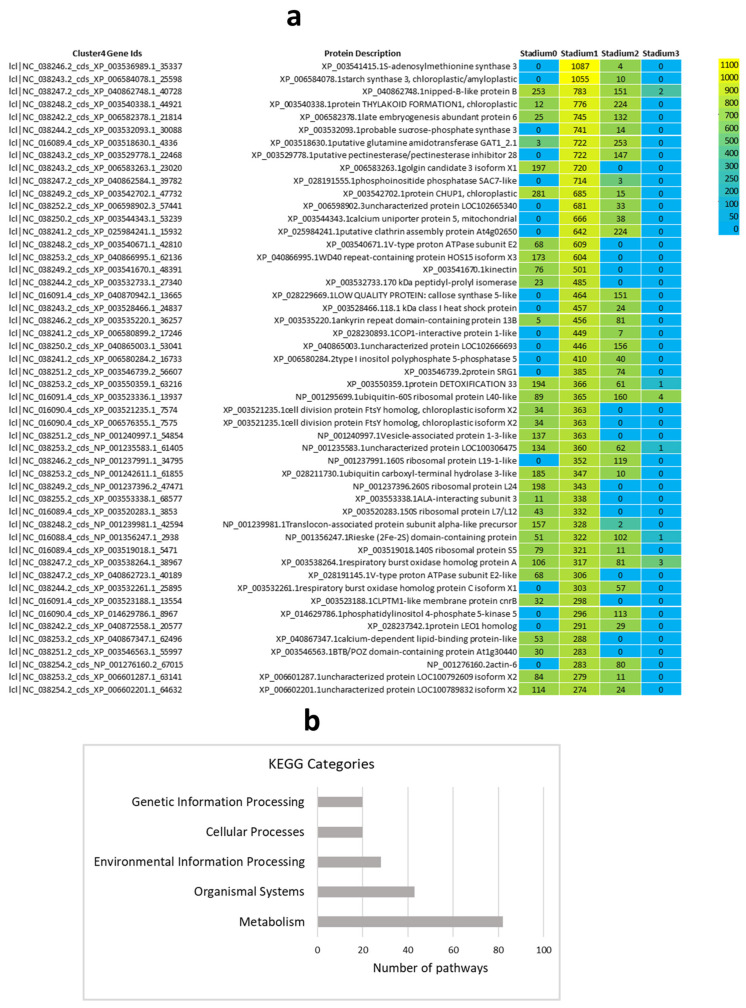
The top 50 DEGs of cluster 4 were visualized in a heatmap, indicating the digital gene expression index (RPM values, (**a**)). The heatmap displays gene expression values derived from three biological replicates pooled. These genes were then mapped to the KEGG database to determine the pathways of main categories (**b**).

The results of the characteristically expressed genes in Cluster 4 are presented in Figure 4, highlighting a pronounced peak in expression at Stadium 1. GO enrichment analysis was performed on the 600 genes in Cluster 4, covering the three major GO categories: biological process (BP), molecular function (MF), and cellular component (CC). Additionally, KEGG pathway categorization was conducted to identify functionally enriched metabolic and signaling pathways. The top 30 significantly enriched GO terms across BP, MF, and CC, as well as key KEGG pathways, are visualized in Figure S1. A corresponding heatmap illustrates the expression patterns of these DEGs (Figure 4a). Using this, 600 gene KEGG pathway analysis was performed and the most significant KEGG categories are presented in Figure 4b.

GO Enrichment analysis suggested that the most enriched BP processes were the regulation of DNA-templated transcription, protein phosphorylation, RNA splicing, and flower development. These findings suggest that key regulatory and developmental pathways are actively engaged during the transition to flowering. The presence of “flower development” directly confirms the dataset’s biological relevance (Figure S1a). MF enrichment analysis revealed significant overrepresentation of terms such as ATP binding, DNA binding, protein kinase activity, and RNA binding. These functions are commonly associated with regulatory proteins, transcription factors, and signaling enzymes, underscoring the importance of gene expression regulation and signal transduction during the floral transition (Figure S1b). CC terms indicated that most DEGs are localized to the nucleus, cytoplasm, and integral components of membranes. This distribution suggests the involvement of both nuclear transcriptional machinery and membrane-associated receptors or signaling proteins in regulating early stadiums of floral development (Figure S1c).

Expression heatmap of cluster-4 displays (Figure 4a) that several gene clusters exhibit high expression only in specific samples, suggesting temporal or tissue-specific roles in floral development. These expression dynamics point to the involvement of tightly coordinated genetic programs, encompassing key transcription factors, hormone-responsive genes, and metabolic enzymes. The following hormone-related genes were found in the heatmap: XP_003541415.1 encodes S-adenosylmethionine synthase 3 (MAT3), an enzyme responsible for the synthesis of S-adenosylmethionine (SAM), which serves as the immediate precursor in the ET biosynthesis pathway. XP_003553338.1—IAA-interacting subunit 3 essential for AUX-dependent gene transcription. XP_006508284.2—Type I inositol polyphosphate 5-phosphatase 5 implicated in ABA and AUX signaling pathways. XP_014697786.1—Phosphatidylinositol 4-phosphate 5-kinase 5 involving in AUX and ABA signal transduction and XP_028211730.1—Ubiquitin carboxyl-terminal hydrolase 3-like involving in AUX and JA hormone responses. The results of the KEGG metabolic pathway analysis (Figure 4b) indicate extensive metabolic reprogramming during flowering. Hormonal signaling and development-related pathways were primarily classified under the “Organismal Systems” category, supporting their central role in coordinating developmental regulation during the floral transition.

### 2.5. Pairwise Analysis of DEGs: Stadium 2 vs. Stadium 0

**Figure 5:**
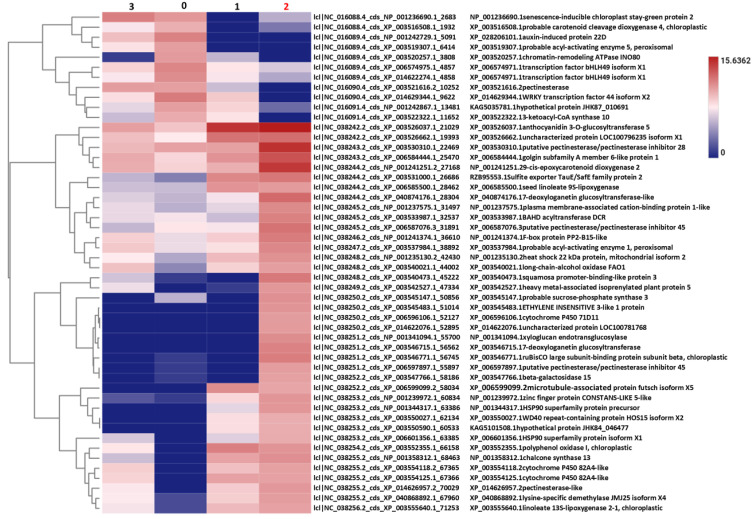
Heatmap showing the expression profiles of genes in Stadium 2, among which we identified a number of hormonal responses across the four experimental conditions (Stadium 0, Stadium 1, Stadium 2, and Stadium 3). Genes associated with AUX response (XP_028206101.1, Auxin-induced protein 22D) and carotenoid cleavage (XP_003516508.1, probable carotenoid cleavage dioxygenase 4) are downregulated in Stadium 2. On the other hand, genes involved in ABA biosynthesis (NP_001241251.2, 12-cis-epoxycarotenoid dioxygenase 2), ET signaling (XP_005515001.1, Ethylene-Insensitive 3-like), and JA pathway (XP_035556401.1, Linoleate 13S-lipoxygenase 2-1) are upregulated in Stadium 2. These expression changes indicate a notable shift in hormonal regulation during Stadium 2, characterized by enhanced ABA, ET, and JA signaling. Concurrently, a downregulation of AUX-responsive genes and carotenoid-related processes was observed. Hierarchical clustering, based on the Euclidean distance between genes, was used to generate the dendrograms displayed on the left. Expression data were calculated using the logarithm of CPM.

The top 50 DEGs from the pairwise comparison between Stadium 2 and Stadium 0 were plotted in a heatmap (Figure 5). Several of these genes are directly involved in hormone biosynthesis, signaling, and response pathways, indicating that substantial hormonal reprogramming occurs at stadium 2. AUX-induced protein 22D (XP_028206101.1) shows a clear downregulation pattern induced by IAA, marking an AUX-sensitive cluster. Carotenoid Cleavage Dioxygenase 4 (CCD4, XP_003516508.1) downregulation and 9-cis-epoxycarotenoid dioxygenase 2 (NCED2, NP_001241251.2) upregulation are involved in ABA biosynthesis, clustering with stress-induced patterns. The upregulation of Ethylene-Insensitive 3-like (EIN3-like, XP_005515001.1) transcription factor in stadium-2 is a prominent marker for ET response in the heatmap. 13S-lipoxygenase 2-1 (LOX, XP_035556401.1) upregulation may trigger the initial steps of the JA pathway.

The flowering-related gene CONSTANS-like 5 (XP_001239972.1) was also identified among the top 50 DEGs. This gene is a key component of the photoperiodic flowering pathway, functioning as a central integrator of light and circadian clock cues. CONSTANS-like 5 promotes the expression of downstream targets such as FLOWERING LOCUS T (FT), thereby facilitating the transition to flowering under appropriate photoperiod conditions. On the heatmap, CONSTANS-like 5 exhibits clear upregulation in both Stadium 1 and Stadium 2, suggesting the activation of photoperiodic flowering signals during these key developmental stadiums. In the heatmap, co-regulation patterns involving NCED2 and EIN3-like genes are also observed alongside CONSTANS-like 5, suggesting potential indirect interactions. NCED2, a key enzyme in ABA biosynthesis, and EIN3-like, a central regulator in ethylene signaling, may modulate flowering through hormone-mediated crosstalk. Their coordinated expression with CONSTANS-like 5 implies a complex regulatory network where hormonal pathways intersect with photoperiodic signaling to fine-tune the timing of floral induction.

### 2.6. Gene Ontology Analysis of DEGs of the Four Investigated Sample Pairs

**Figure 6:**
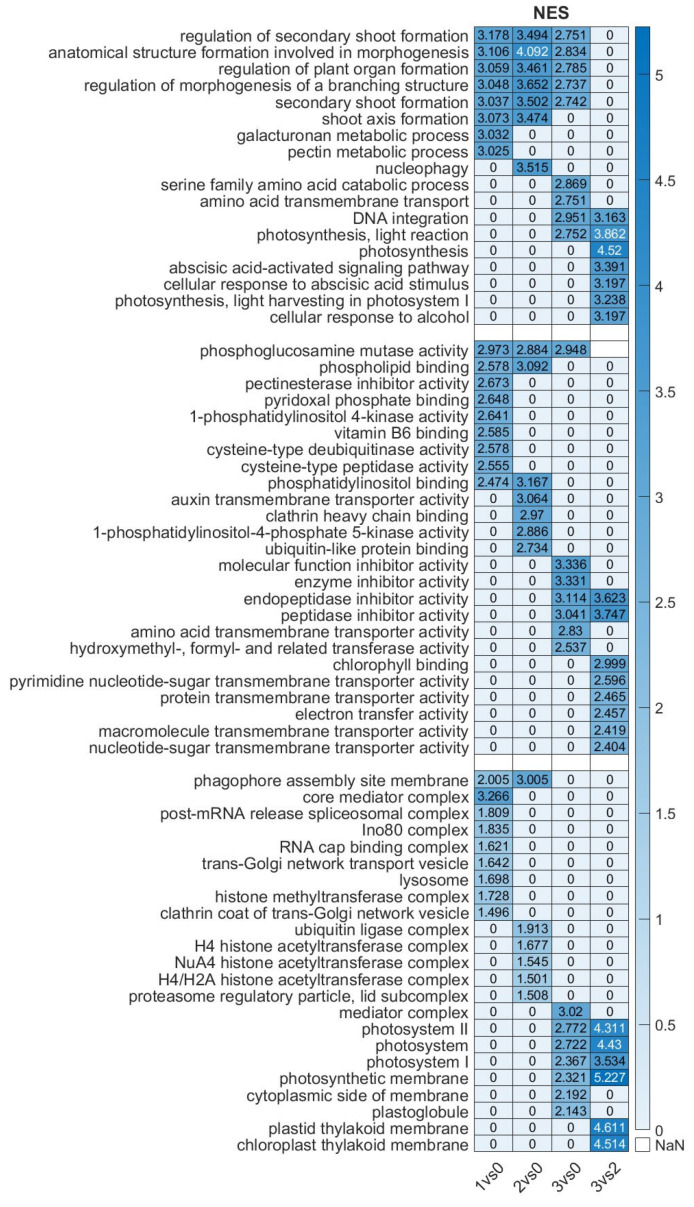
Heatmap of enriched GO terms of differentially expressed genes in the investigated floral stadiums. The ranking was performed based on NES (normalized enrichment score) values, which represent the enrichment score for each gene set after normalization across all analyzed gene sets. NaN means no data available. The NES data scale is shown on the right.

We investigated the GO categories across the samples (Figure 6). The biological process (BP) category revealed strong enrichment for terms related to plant morphogenesis and organ formation in the comparisons of Stadium 1 vs. Stadium 0 and Stadium 2 vs. Stadium 0. Biological process analysis revealed that photosynthesis-related processes were significantly enriched in the Stadium 3 vs. Stadium 0 comparison, indicating increased transcriptional activity of photosynthetic genes in Stadium 3. This pattern implies that, in the overblown or post-anthesis flower, cellular response to ABA and signaling was characteristic at the end of flower ripening in the sample pair Stadium 3 vs. 2. In the molecular function (MF) category, chlorophyll binding and membrane transport activity were notably enriched in the Stadium 3 vs. Stadium 0 comparison, indicating that these functions are characteristic of the overblown flower stadium. In contrast, auxin transporter activity was specifically enriched in the Stadium 2 vs. Stadium 0 comparison, coinciding with the peak of floral development. Furthermore, in the overblown flower stadium (Stadium 3 vs. Stadium 0), several GO terms associated with enzyme inhibitor activity were enriched. In contrast, during the early flowering stadium (Stadium 1 vs. Stadium 0), significant enrichment of GO terms related to amino acid metabolism, such as pyridoxal phosphate binding and vitamin B6 binding, was observed. These functions are closely associated with phytohormone biosynthesis and stress response pathways, indicating heightened hormonal regulation and metabolic adjustment as the plant transitions to reproductive development. In the cellular component category, the chloroplast-related GO names are characteristic only in the overblown stadium (3 vs. 0 and 3 vs. 2). Histone modification-related processes were significantly enriched in the Stadium 2 vs. Stadium 0 comparison, indicating active epigenetic regulation during this key developmental stage. The presence of these processes suggests a high level of transcriptional reprogramming at Stadium 2, coinciding with peak flowering activity. GO terms associated with autophagy were identified in both the early (Stadium 1 vs. Stadium 0) and late (Stadium 3 vs. Stadium 0) flowering stadium comparisons. This suggests that autophagic processes may play a crucial role in maintaining cellular homeostasis and recycling resources during these pivotal developmental transitions. The presence of autophagy-related activity at these stadiums implies a potential regulatory mechanism influencing hormone balance, thereby affecting the timing and induction of flowering. The GO names referred to hormonal regulation and signaling; however, it is not clear which hormone has the most important regulatory role at different flowering stages. Therefore, we performed a pathway analysis combined with gene expression analysis, focusing on plant hormone signaling pathways.

### 2.7. Gene Set Enrichment Analysis (GSEA) Across the Four Investigated Sample Pairs, Focusing on Phytohormone-Related Genes

**Figure 7:**
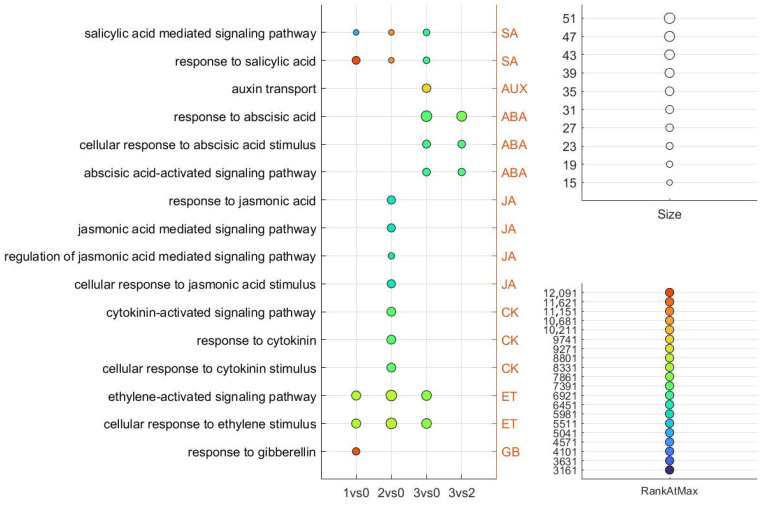
The phytohormone-related GO terms identified in the GSEA analysis of the investigated sample pairs are shown. The size of each term represents the number of DEGs, while RankAtMax indicates the point in the ranked gene list where the gene set reaches its maximum enrichment. Higher RankAtMax values suggest stronger collective gene expression within the set. Adjusted *p*-values (FDR) for GO term enrichment are represented in Table S1. All GO terms shown in the figure have FDR-adjusted *p*-values < 0.05, indicating statistically significant enrichment.

We performed GSEA to evaluate predefined gene sets exhibiting statistically significant expression differences between the investigated floral developmental stages. Gene ranking was determined using the maximum enrichment score to identify gene sets that were either upregulated or downregulated. This approach enabled us to gain insights into the biological processes most strongly linked to specific flowering phenotypes. Subsequently, we focused specifically on phytohormone-associated GO terms identified within the enriched gene sets across the investigated sample pairs (Figure 7). Upon summarizing the results, we found that SA- and ET-associated GO terms were consistently enriched across all floral developmental stadium comparisons (1 vs. 0, 2 vs. 0, and 3 vs. 0), suggesting their broad involvement throughout flower development. In contrast, a single AUX-associated GO term was uniquely identified in the 3 vs. 0 comparison, indicating a more stadium-specific role for auxin signaling at the overblown flower stadium.

JA-associated GO terms were exclusively enriched in the 2 vs. 0 comparison, a pattern that was also observed for CK-associated GO terms, suggesting a specific role for these hormones during peak floral development. Interestingly, GA-associated GO enrichment was detected only in the 1 vs. 0 comparison, indicating that GA signaling may play a more prominent role during the early transition from vegetative to reproductive growth.

### 2.8. Expression Involved in Plant Hormone Signal Transduction Across the Four Investigated Floral Stadiums

**Figure 8:**
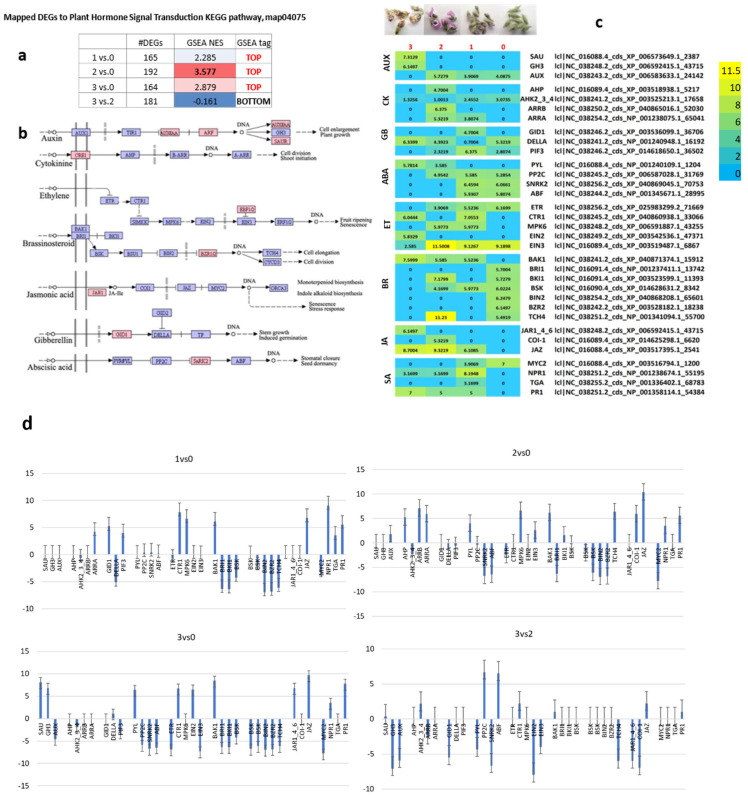
Mapped DEGs to the plant hormone signal transduction KEGG pathway. The number of mapped DEGs and normalized enrichment score (NES) of gene set enrichment analysis (GSEA) of the pathway in the samples are summarized in (**a**) image. We determined that mapped gene sets are overrepresented at the top or bottom of the gene-ranked list in the expression dataset (**a**). The pathway diagram, which indicates the mapped DEGs, is visualized in image (**b**). Expression patterns of mapped DEGs in the four investigated samples are visualized in a heatmap (**c**) indicating the RPM digital gene expression index that were validated by RT-qPCR analysis (**d**).

Following the mapping of sequences to the KEGG pathway map04075, we analyzed the DEGs associated with plant hormone signal transduction. Changes were observed in nearly all genes mapped to this pathway. The highest number of mapped DEGs was identified in the comparison between Stadium 2 and Stadium 0, while a downregulation of the pathway was observed exclusively in the Stadium 3 vs. Stadium 2 comparison (Figure 8a). To further understand these trends, we examined the expression profiles of the most significantly regulated DEGs across the four developmental stadiums. After mapping the entire transcript set to this pathway (Figure 8b), DEGs were determined across the samples. Their expression patterns were analyzed in silico and validated through RT-qPCR analysis (Figure 8c,d). In the case of AUX hormonal signaling, differential expression of genes was observed in Stadium 3, including upregulation of SAU and GH3 and downregulation of AUX genes. CK signaling changes were most characteristic in Stadium 2, characterized by upregulation of *AHP*, *ARRA*, and *ARRB* genes, along with downregulation of *AHK2/3/4*. In gibberellin (GA) signaling, DEGs were upregulated in Stadium 1, including *GID1*, *DELLA*, and *PIF3* genes. Expression changes in ABA signaling genes were characteristic of Stadiums 2 and 3, with upregulation of *PYL* and downregulation of *PP2C*, *SnRK2*, and *ABF* genes observed across all investigated stadiums. Upregulation of *ETR* and *EIN3*, along with downregulation of *EIN2*, was detected in Stadiums 0, 1, and 2. Additionally, *MPK6* was upregulated in Stadiums 1 and 2. CTR was upregulated in Stadiums1 and 3. Genes involved in the BR signaling pathway showed increased expression in Stadium 0, including *BRI1*, *BAK1*, *BIN2*, and *BZR2*; however, *BAK1* was downregulated in this stadium. Notably, *TCH4* exhibited remarkably high expression in Stadium 2. In JA signaling, repression of *JAR1/4/6* genes was observed in all investigated stadiums, except in Stadium 3, where these genes were not repressed. *COI1* expression was detected exclusively in Stadium 2, while *JAZ* was downregulated in Stadium 0. Notably, no JA signaling-related genes were expressed during Stadium 0. In SA signaling, the MYC2 gene showed upregulation in Stadiums 0 and 1. The highest expression level of NPR1 was observed in Stadium 1; however, this gene was upregulated in all investigated stadiums except Stadium 0. TGA expression was characteristic of Stadium 1. Additionally, PR1 was expressed in all mature samples (Stadiums 1 to 3), but not in Stadium 0.

### 2.9. Expression Analysis of MADS-Box Genes During Floral Development Across the Four Investigated Stadiums

**Figure 9:**
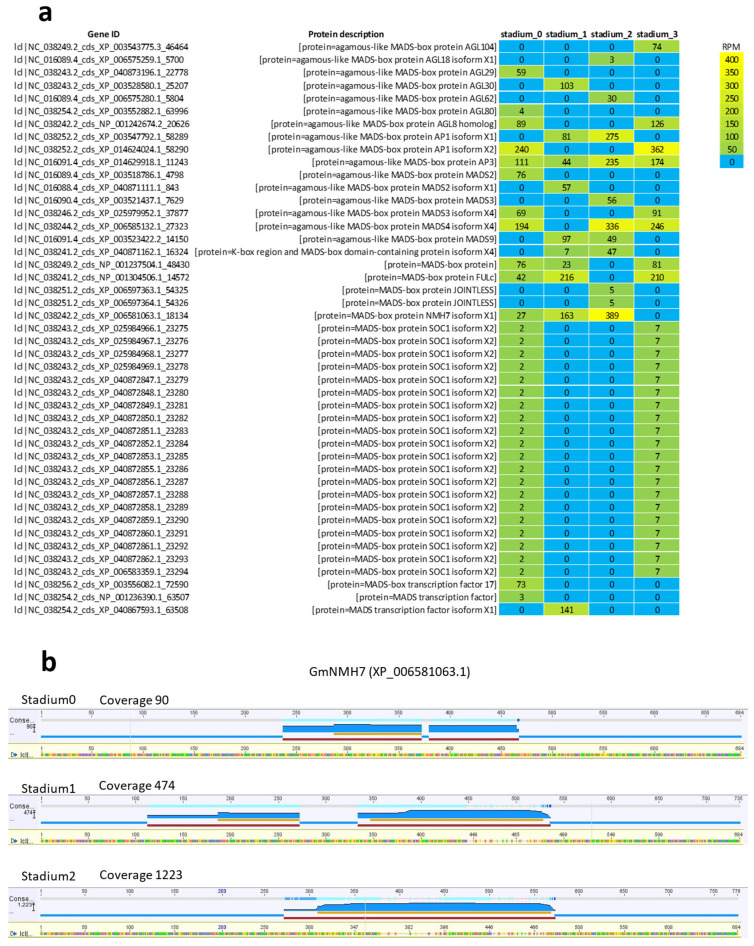
Heatmap of MADS-box DEGs expressed in the investigated floral tissues (**a**). Gene expression patterns were analyzed based on digital gene expression values, measured in Reads Per Million (RPM). Panel (**b**) visualizes read abundance mapped to the *GmNMH7* gene, with the highest coverage observed in Stadium 2. Read abundance—reflecting gene expression levels—showed a progressive increase across floral development stadiums, indicating upregulation.

MADS-box genes in soybean play a fundamental role in regulating floral organ development and flowering time. Their regulation provides opportunities to optimize soybean yield and adaptability to diverse environmental conditions. To investigate their expression, we retrieved all MADS-box CDSs from the *G. max* genome and calculated their expression levels using RPM digital normalization. A total of 110 MADS-box gene sequences were identified, of which 45 showed detectable expression in the investigated samples (Figure 9a). The *JOINTLESS* gene, which regulates the formation of the abscission zone, was expressed in Stadium 2. *SOC1*, a known inducer of early flowering whose reduced activity can delay flowering, was expressed in the two extreme stadiums—Stadium 0 and Stadium 3. Interestingly, the root-specific gene *NMH7* was expressed in Stadiums 0, 1, and 2, with the highest RPM value observed in Stadium 2. Although *NMH7* is primarily involved in root and tuber development, and there is no direct evidence linking it to flowering in soybean, the expression of *GmNMH7* in floral tissues has not been previously reported. Visualization of read abundances mapped to the GmNMH7 gene revealed the highest coverage at developmental Stadium 2. The temporal distribution of mapped reads, reflecting gene expression levels, indicated a progressive upregulation over the course of floral development (Figure 9b).

## 3. Discussion

### 3.1. Reference-Guided Transcriptome Assembly and Stadium-Specific Differential Gene Expression Analysis During Soybean Floral Development

The present study focuses on the reference-guided transcriptome assembly of soybean flower development across four distinct developmental stadiums, followed by comprehensive gene re-annotation. This approach establishes a robust framework for investigating gene expression dynamics throughout floral ontogeny. High-throughput RNA sequencing using Illumina technology yielded high-quality reads, facilitating reliable mapping to the *G. max* reference genome. The mean library size of approximately 2 million reads per sample ensured adequate coverage for transcriptome-wide analyses. The annotation process demonstrated a high success rate, particularly in plant systems, thereby supporting the accuracy and completeness of downstream functional analyses. While several previous studies have provided detailed insights into transcriptomic alterations associated with soybean flower development and may serve as valuable references for evaluating RNA-Seq-based annotation rates and gene expression profiles [53,54,55,56], the present study represents the first temporal analysis of reference-guided transcriptome assembly and differential gene expression related to floral development under field-grown conditions.

The substantial number of DEGs identified across the developmental stadiums of soybean flowers reflects a dynamic reprogramming of transcriptional activity during floral maturation. The highest number of DEGs was observed in the comparison between Stadium 1 and Stadium 0, suggesting that the early phase of flower development involves the most dramatic transcriptional shifts. This is consistent with the initiation of key developmental and morphogenetic processes that likely require extensive gene activation and repression [57,58].

In contrast, the comparison between Stadium 3 and Stadium 0 yielded the lowest number of uniquely upregulated genes, indicating that gene expression becomes more stabilized at later stadiums, possibly reflecting the culmination of developmental programs and the transition toward reproductive maturity. Notably, the largest number of uniquely downregulated genes was found in the Stadium 3 vs. Stadium 2 comparison, suggesting a strong transcriptional suppression as the flower transitions from maturation to final developmental arrest or senescence [59]. This downregulation may be associated with the cessation of cellular processes no longer required or with the activation of programmed cell death pathways [60].

### 3.2. Functional Characterization of Cluster-4 Reveals Hormonal and Transcriptional Control of Early Floral Morphogenesis in Soybean

To gain deeper insights into the biological processes underlying early flower development, we performed a functional analysis of the 600 genes in Cluster 4, which exhibited peak expression in Stadium 1. GO enrichment analysis revealed a significant overrepresentation of terms related to organ development, cell division, hormone-mediated signaling pathways, and transcription factor activity. These findings support the hypothesis that Stadium 1 represents a critical window for floral organ specification and early morphogenesis. More specifically, biological process GO terms such as regulation of meristem development, floral whorl morphogenesis, and auxin-activated signaling pathway were among the most enriched, suggesting that Cluster 4 genes are tightly linked to the initiation and spatial organization of floral organs. The enrichment of auxin and gibberellin-related signaling components is consistent with previous studies indicating the essential roles of these hormones in early flower patterning [61].

In terms of molecular function, a substantial number of genes were associated with DNA-binding transcription factor activity, including members of the MADS-box, bHLH, and MYB families, which are known to regulate floral identity and meristem fate [62]. Additionally, the presence of genes related to chromatin remodeling and RNA processing points to transcriptional and post-transcriptional regulatory mechanisms being activated early in development [63].

KEGG Pathway analysis further revealed enrichment in protein ubiquitination, cell cycle regulation, and cell wall modification pathways, highlighting the importance of controlled cellular proliferation and expansion during the early morphogenetic phase [64].

Collectivelyr, the temporal expression pattern and enriched functional categories of Cluster 4 underscore its central role in orchestrating the developmental shift from vegetative to reproductive growth. The coordinated expression of regulatory genes and hormonal pathways suggests that this cluster contributes to the precise establishment of floral organ identity and positioning in soybean.

### 3.3. Transition from Auxin-Dominated Processes to Stress-Associated Pathways in Soybean Flower Development at Stadium 2

The pairwise comparison between Stadium 2 and Stadium 0 revealed a distinct transcriptional landscape characterized by extensive hormonal reprogramming. Several key genes among the Top50 DEGs highlight the dynamic interplay of phytohormones at this stadium of flower development, indicating a transition point in regulatory control toward floral maturation.

Notably, the clear downregulation of Auxin-induced protein 22D suggests a reduction in auxin sensitivity or auxin signaling activity in Stadium 2. As auxin is known to play critical roles in organ initiation and early floral patterning [65], its repression at this stadium may reflect the culmination of these early developmental events and a shift toward other regulatory pathways. This is further supported by the simultaneous downregulation of *CCD4* and the upregulation of *NCED2*—two antagonistic components in the carotenoid metabolic pathway [66]. While *CCD4* participates in carotenoid catabolism, *NCED2* is a rate-limiting enzyme in ABA biosynthesis. The observed increase in *NCED2* expression suggests enhanced ABA production in Stadium 2, potentially linked to developmental regulation and/or stress adaptation mechanisms.

The upregulation of *EIN3*-like, a key transcription factor in ET signaling, reinforces the notion that stress-related hormonal pathways become more prominent during Stadium 2. Ethylene, often acting synergistically with ABA and antagonistically with auxin [67], is known to influence floral organ maturation and senescence processes [33]. In parallel, the induction of *LOX*, a marker of JA biosynthesis, further supports the activation of stress- and defense-related pathways at this stadium. The coordinated upregulation of ABA, ET, and JA pathways suggests a complex hormonal network that might help fine-tune the balance between growth and developmental transitions in flower formation [68].

Importantly, the presence and upregulation of *CONSTANS-like 5* in Stadium 1 and Stadium 2 point to the possible onset of photoperiodic flowering signals during these stadiums. CO acts as a central integrator of circadian and light cues, promoting the expression of FT, a key florigen gene [69,70]. The expression dynamics of *CONSTANS-like 5*, in conjunction with the activity of *NCED2* and *EIN3*-like, may reflect a hormonal crosstalk that modulates the photoperiod pathway. While no study directly links the interaction of these three genes, the complexity of hormone crosstalk and transcriptional regulation in plants suggests that *CO, NCED2*, and *EIN3* may interact indirectly through broader signaling networks. While GAs can enhance CO function in long-day plants, hormones like ABA and ET are known to negatively influence CO-mediated flowering, either by delaying floral transition or suppressing *FT* expression [71,72,73,74].

Collectively, the expression patterns observed in Stadium 2 reveal a complex shift in hormonal dominance—from early auxin-driven developmental processes to stress-associated ABA, JA, and ET signaling. Simultaneously, the activation of flowering-related regulators such as *CONSTANS-like 5* suggests that this stadium may serve as a critical transitional phase where environmental and endogenous signals converge to determine the timing and progression of reproductive development in soybean flowers.

### 3.4. Coordinated Auxin, ABA, and Epigenetic Reprogramming Define Distinct Phases of Soybean Floral Maturation

The GO enrichment and GSEA analyses revealed stadium-specific functional transitions in the soybean floral developmental process, underlining the coordinated action of metabolic, hormonal, and gene regulatory mechanisms. These patterns emphasize the complexity and dynamism of the flower maturation process, which appears tightly regulated by both developmental cues and stress-related signals [75,76,77,78,79,80].

In the early and mid-flowering stadiums (Stadium 1 and Stadium 2), GO categories in the biological process were dominated by terms related to morphogenesis, organ formation, and amino acid metabolism. The overrepresentation of pyridoxal phosphate and vitamin B6 binding in Stadium 1 supports a role for amino acid metabolism not only in primary metabolism but also in hormone biosynthesis (e.g., ethylene, auxin, and cytokinin precursors), suggesting a metabolically active state that prepares the plant for reproductive success [81,82,83]. The presence of autophagy-related GO terms in both Stadium 1 and Stadium 3 also suggests substantial cellular remodeling and resource reallocation processes, which could influence hormonal balances that determine floral timing [84,85,86].

Stadium 2 was particularly enriched in processes associated with histone modification and epigenetic regulation, implying intense transcriptional reprogramming during the peak of flower development. This is in line with the high number of differentially expressed genes mapped to the plant hormone signal transduction pathway in this stadium, indicating a regulatory pivot point where multiple hormone signals converge to fine-tune floral development [87,88].

By contrast, Stadium 3 (the overblown flower) showed a metabolic shift, enriching photosynthesis-related processes, chloroplast-related components, and enzyme inhibitor activities. The chlorophyll-binding GO terms and increased membrane transport activity suggest a metabolic “rebalancing” as the flower completes its functional role. This stadium was also characterized by ABA-associated responses, pointing to the involvement of ABA in the later stadiums of flower senescence or desiccation preparation [89,90].

Hormone-related GO terms confirmed the stadium-specific involvement of distinct hormonal pathways. GSEA results demonstrated that SA and ET signaling were active across all stadiums, underscoring their roles in basal floral development and stress signaling. In contrast, auxin- and cytokinin-related GO terms were specific to Stadium 2, consistent with their known roles in floral organ formation and cell proliferation [51,91]. JA signaling was only detected in Stadium 2, likely representing a transient activation that supports reproductive tissue differentiation and defense readiness [92,93]. Notably, BR-related signaling appeared to dominate Stadium 0, indicating a preparatory growth phase. GA-related terms were found only in Stadium 1, supporting its proposed role in the initiation of flowering [94,95,96,97].

Mapping of DEGs to the KEGG pathway of plant hormone signal transduction provided further resolution into the activity of specific hormone signaling components. For instance, auxin-related DEGs (SAUR, GH3, AUX/IAA) displayed a dynamic pattern in Stadium 3, suggesting regulatory feedback as the flower approaches senescence. Through changes in AHKs, AHP, and ARRs, cytokinin signaling was predominantly altered in Stadium 2, reflecting a peak in cell division and organ differentiation. ABA-related signaling (PYL, PP2C, SnRK2) was clearly activated in Stadiums2 and 3, which corresponds well with stress signaling and developmental transitions. ET-related components (ETR, EIN3, CTR) showed consistent upregulation across multiple stadiums, confirming their continuous role in flower maturation and possibly senescence.

SA pathway markers such as *NPR1*, *TGA*, and *PR1* exhibited strong expression in the mid- to late-floral stadiums, suggesting activation of defense priming in reproductive tissues [98,99,100]. Interestingly, the BR signaling components (*BIN2*, *BZR2*) were mostly enriched in Stadium 0, supporting their role in promoting floral transition via cell expansion mechanisms [94]. In contrast, the late JA signaling gene COI1 was expressed explicitly in Stadium 2, and repressed JAZ expression in earlier stadiums supports a release of JA-mediated transcriptional repression [101,102].

Together, these multi-layered analyses indicate that a complex, stadium-dependent reorganization of hormone signaling networks regulates the progression of floral development in soybean. Stadium 2 emerges as a central developmental hub characterized by epigenetic activity, cytokinin/JA responsiveness, and dynamic auxin and GA signaling, while Stadium 3 reflects a shift toward stress-related ABA and ET signaling, possibly associated with flower senescence and functional decline.

### 3.5. Stadium-Specific Expression of MADS-Box Transcription Factors and Novel Candidate Gene GmNMH7 Reveals Complex Regulatory Networks in Soybean Flower Development

Our study identified 45 MADS-box genes expressed across four developmental stadiums of soybean flowers, reflecting a broad functional engagement of this gene family.

The expression of *JOINTLESS* in Stadium 2 is of particular interest. *JOINTLESS* is known from tomato and other species as a regulator of the abscission zone formation and inflorescence architecture [103]. Its peak expression during the flowering stadium in soybean suggests a possible role in preparing for organ separation or tissue remodeling that occurs post-anthesis, although its specific function in legumes remains experimentally validated.

We also observed the expression of *SOC1* in Stadium 0 and Stadium 3. As a central integrator of multiple flowering signals, *SOC1* promotes floral transition by regulating downstream genes such as *LEAFY* and *APETALA1*. Its early expression in Stadium 0 could be linked to the initiation of reproductive development. In contrast, its expression in Stadium 3 might reflect regulatory feedback or a role in floral organ senescence and late-stadium morphogenesis.

One of the most unexpected findings was the strong expression of *GmNMH7*, a gene annotated as root-specific, across floral tissues, particularly peaking in Stadium 2. *NMH7* has been previously described as a membrane hydrophobic protein involved in nodule formation and root tissue development in legumes. Its expression in floral tissues has not been reported before, and our results—showing high transcript abundance and mapped read coverage—indicate that *GmNMH7* might have an uncharacterized function in reproductive organ development. This may reflect an evolutionary co-option of root-associated genes into new developmental contexts, a phenomenon increasingly reported in plant evolution. The possibility that *GmNMH7* contributes to cell wall remodeling, transport processes, or signaling in floral tissues during peak flowering merits further study. Future work should address whether these gene expression patterns are conserved across cultivars and environmental conditions, and how they contribute to floral determinacy, fertility, and ultimately yield in soybean.

Altogether, these results highlight the complexity of transcriptional regulation during floral development in soybean. The identification of both canonical and unexpected regulators such as *SOC1*, *JOINTLESS*, and *GmNMH7* underscores the need for deeper functional analyses.

### 3.6. Proposed Model and Breeding Implications

Based on the transcriptomic patterns observed, we propose a three-phase regulatory model of soybean flower development: (i) early activation of gibberellin, cytokinin, and chromatin modifiers (S0–S1), (ii) auxin- and jasmonate-driven anthesis peak with enriched signal transduction and protein turnover (S2), and (iii) ABA-mediated floral aging and photosynthetic reprogramming (S3). This model aligns with known physiological transitions and provides a framework for exploring gene–hormone interactions during flowering.

These findings hold potential relevance for molecular breeding efforts. By targeting key regulators identified in this study—such as *ARR-A* cytokinin responders, *EIN3*-like ethylene mediators, and *SOC1* homologs—breeders could modulate flowering timing, synchrony, or resilience under environmental stress. The hormone-responsive clusters and DEG datasets are valuable for predicting floral traits in soybean and related crops.

### 3.7. Limitations and Future Directions

A limitation of this study is the relatively low sequencing depth of the pooled libraries. Although qPCR validation supports the reliability of key findings, this limited depth may reduce the ability to capture low-abundance transcripts.

While the dataset offers high-resolution temporal coverage, it is limited to a single soybean genotype and short-read sequencing. Future studies using long-read, single-cell, or spatial transcriptomics could provide a finer insight into isoform diversity and tissue-specific expression. Furthermore, directly quantifying phytohormones and including proteomic data would strengthen the link between transcript abundance and physiological states. Functional validation of candidate genes, especially *GmNMH7* and less-characterized MADS-box members, remains an important next step in translating transcriptomic signatures into phenotypic understanding.

## 4. Materials and Methods

### 4.1. Plant Materials and RNA-Sequencing

A collection of the four stadiums of *G. max* flower samples was reported in our previous study published in the *Data in Brief* journal [56]. The soybean cultivar ES Director was used in field cultivation. Total RNA from the three biological replicates per stadium was pooled in equimolar amounts prior to library preparation.

The four stadiums of *G. max* flowers were collected in liquid nitrogen and were stored at −80 °C. The total RNA from flower tissues was isolated using the TaKaRa Plant RNA Extraction Kit, following the manufacturer’s instructions (Takara Bio Inc.; Shiga, Japan). The concentration and purity of the RNA sample were assessed using the Qubit 4 Fluorometer (Thermo Fisher Scientific; Waltham, MA, USA) and the Agilent TapeStation system (Agilent Technologies Inc.; Santa Clara, CA, USA). For the experiment, 2.0 g of total RNA was used as the starting material for cDNA synthesis and library construction. The TruSeq RNA Sample Preparation Kit was used to supplement the mRNA synthesis, cDNA synthesis, and prepare the library for Illumina NextSeq (San Diego, CA, USA) sequencing.

75-bp single-end reads were generated and aligned to the high-quality soybean reference genome, enabling the efficient and unambiguous mapping of the majority of reads to annotated genes, with a focus on general transcriptomic profiling.

Illumina RNA-seq reads of the four distinct stadiums of flowers were deposited in the NCBI Sequence Read Archive (SRA) under the following accession numbers: SRR18059506, SRR18059505, SRR18059504, SRR18059503. The BioProject can be found under the following accession: PRJNA807844. Reference guided assembly was performed using these reported reads.

### 4.2. Reference-Guided Assembly and Re-Annotation of Mapped Genes

The processed reads were mapped to the *G. max* reference genome (GCF_000004515.6) obtained from NCBI on 11 November 2024. Bowtie2 was applied for read alignment. The reads that matched the reference genome CDS were retained for further analysis. The CDS sequences were reannotated, including Blastx alignment, Gene Ontology (GO) mapping, GO annotation, and functional analysis (GO-Slim). Blast alignments were performed against the NR database (version dated 23 January 2025), utilizing the taxonomic filters for Viridiplantae (NCBI:txid33090). This mapping was automated using OmicsBox (Bioinformatics Made Easy, BioBam Bioinformatics, https://www.biobam.com/omicsbox, accessed on 3 January 2025).

### 4.3. Analysis of Differentially Expressed Genes (DEGs)

Sequencing reads were aligned to quantify gene expression from RNA-Seq data, with the results annotated in relation to genomic features. A count table (abundance matrix) was constructed for the CDS sequences to facilitate downstream analysis. To identify genes with significantly different expressions between experimental groups, pairwise differential expression analysis was performed using DESeq, a Bioconductor package (version 3.20). The experimental set-up of pairs was based on the following:

Stadium 1 vs. Stadium 0 allows the identification of genes associated with the earliest morphological changes following floral meristem establishment, including initiation of organ primordia.

Stadium 2 vs. Stadium 0 and Stadium 3 vs. Stadium 0, to capture progressively larger transcriptional changes relative to the undifferentiated meristematic state (Stadium 0), allowing the identification of stage-specific and developmentally induced genes.

Stadium 3 vs. Stadium 2, to highlight gene expression changes associated with the transition from peak anthesis to the onset of floral senescence.

Using Stadium 0 as a baseline reference for multiple comparisons provided a consistent framework to assess transcriptional reprogramming throughout floral development.

The Stadium 3 vs. 2 comparison was included to dissect late-stage regulatory changes that are not detectable when compared only to early developmental stages. This stepwise design allowed us to detect both gradual and stage-specific gene expression dynamics, which is essential for understanding the coordination of floral organ differentiation and maturation.

Top50 pairwise DEGs were displayed in a heatmap indicating the hierarchical clustering, based on the Euclidean distance between genes. Expression data were determined using the logarithm of CPM. This differential expression analysis was combined with enrichment analysis of the examined sample pairs. The Generalized Linear Model (GLM) quasi-likelihood F-test was applied in the analysis of the DEGs.

### 4.4. Gene Set Enrichment Analysis (GSEA)

GSEA was performed to analyze the pairwise sample comparisons [104]. We ranked gene sets based on their upregulation or downregulation to identify those most associated with the investigated flowering phenotypes and to gain insights into the biological processes involved. GSEA results were evaluated using the RankatMax Score. The RankAtMax score refers to a specific metric used to evaluate the correlation between gene sets and the ranked list of genes based on their differential expression. The score is calculated at the position where the gene set achieves its maximum enrichment in the ranked list. A higher RankAtMax score suggests a stronger and more significant relationship between the gene set and the observed phenotype. For the analysis of the ranked gene list of DEGs, the following formula was applied: Rank = sign(logFC) × (−log10(*p*-Value)), where logFC represents the log2-fold change in expression between conditions.

### 4.5. KEGG Pathway Analysis

Pathway analysis was conducted using the KEGG database to gain an overview of the biological mechanisms associated with the pairwise DEGs and to summarize the molecular mechanisms underlying the data. We mapped the annotated sequences of GSEA pairwise DEGs to relevant pathways, performing an intermediate step to match each gene product with the most probable candidate in the pathway database. The mapping process was automated using OmicsBox (Bioinformatics Made Easy, BioBam Bioinformatics, https://www.biobam.com/omicsbox, accessed on 10 January 2025).

### 4.6. Digital Gene Expression Analysis (RPM)

Genes mapped to the “plant signal transduction” KEGG pathway and expressed MADS-box genes mapped to the *G. max* genome were selected (GOIs) to determine digital gene expression values. The Reads Per Million (RPM) mapped read values for these GOIs were determined and compared across samples.

### 4.7. Gene Expression Analysis by RT-qPCR

RT-qPCR analysis was performed with genes mapped to the plant hormone signal transduction KEGG pathway. Total RNA was quantified using a Nanodrop 2000c spectrophotometer and a Qubit 2.0 fluorometer (Thermo Fischer Scientific, Waltham, MA, USA). Five hundred nanograms (ng) of total RNA was reverse transcribed using the M-MuLV RT (Maxima First Strand cDNA Synthesis Kit, Thermo Fischer Scientific, Waltham, MA, USA). A total of 50 ng of cDNA was used in 10 μL reactions for real-time PCR using the Xceed qPCR SG 2× Mix (Institute of Applied Biotechnologies, Praha-Strašnice, Czech Republic) and a CFX384 Touch Real-Time PCR Detection System (Bio-Rad, Hercules, CA, USA). The data were analyzed using the ΔΔCt method [105]. Relative gene expression was determined for GAPDH and TUBB3 reference genes. Values are mean ± SEM (*n* = 4). *p* < 0.05 vs. control, *p* < 0.05 vs. treated group.

## 5. Conclusions

The molecular regulation of soybean floral development involves complex hormonal signaling and transcriptional control networks. In this study, we performed a reference-guided transcriptome assembly and differential gene expression analysis across four developmental stadiums of field-grown soybean flowers using high-throughput Illumina RNA-Seq data. Our results revealed dynamic transcriptional reprogramming, with the most pronounced changes occurring at the early transition (Stadium 1 vs. Stadium 0). The phytohormone signaling pathways showed stadium-specific activation: organogenesis-related auxin, gibberellin, and cytokinin signaling were predominant in Stadium 1, while Stadium 2 exhibited a major hormonal shift with upregulation of abscisic acid, ethylene, and jasmonic acid pathways. This stadium appears to serve as a central regulatory node in floral maturation. The expression of *CONSTANS-like 5* during this transition suggests the integration of photoperiodic cues into hormonal regulation. Notably, the root-specific *GmNMH7* gene showed unexpectedly high expression in floral tissues, especially in Stadium 2, implying a possible novel function. These findings provide new insights into the temporal coordination of hormonal pathways during flower development and highlight the potential of stress-associated signals in shaping reproductive transitions in soybean.

## Data Availability

Data are available in the NCBI Sequence Read Archive (SRA) under the following accession numbers: SRR18059506, SRR18059505, SRR18059504, SRR18059503. The BioProject can be found under the following accession: PRJNA807844.

## Supplementary Materials

The supporting information can be downloaded at https://www.mdpi.com/article/10.3390/ijms26136455/s1.
