# Supplementary material for: Temporal Shifts in Hormone Signaling Networks Orchestrate Soybean Floral Development Under Field Conditions: An RNA-Seq Study
Source: Int J Mol Sci. 2025 Jul 4;26(13):6455. doi: 10.3390/ijms26136455 (PMC12250341; doi:10.3390/ijms26136455)
Supplement: Supplementary file 1 [file ijms-26-06455-s001.zip › ijms-3675898-supplementary.pdf]

Figure S1

Analysis of genes of Cluster-4. The 600 genes of Cluster-4 were selected to determine the top 30 GO categories involving biological process (a), molecular function (b), and cellular component (c).

a

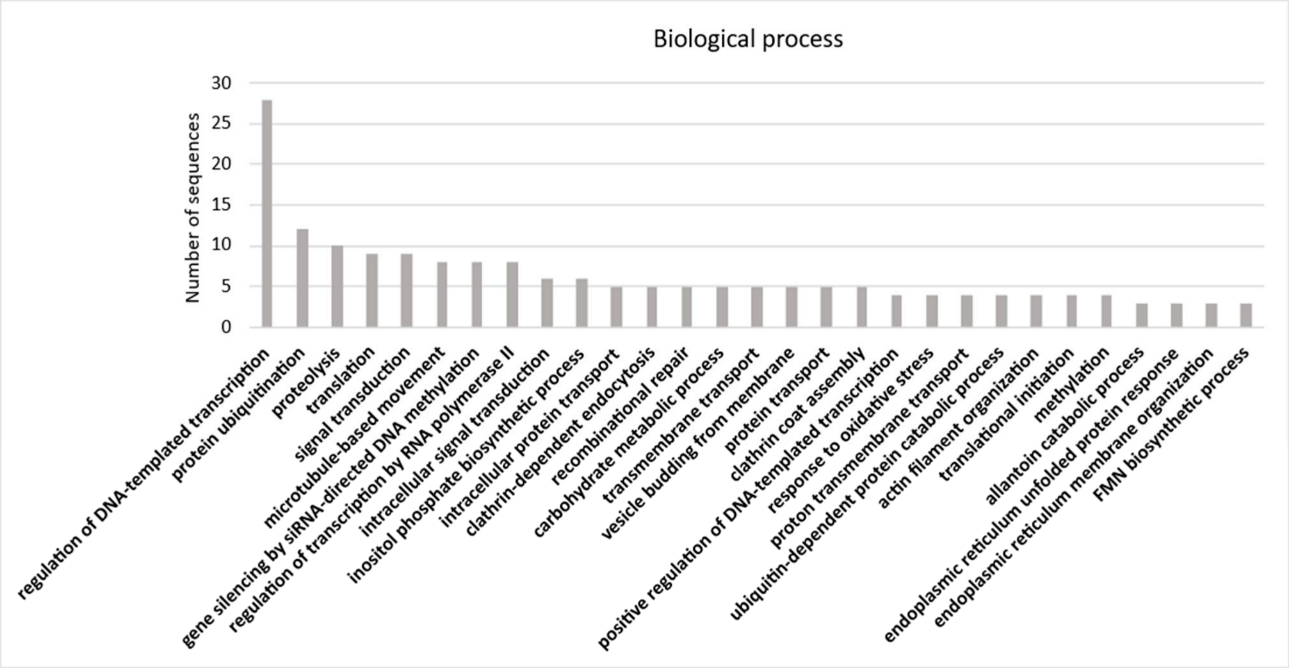

b

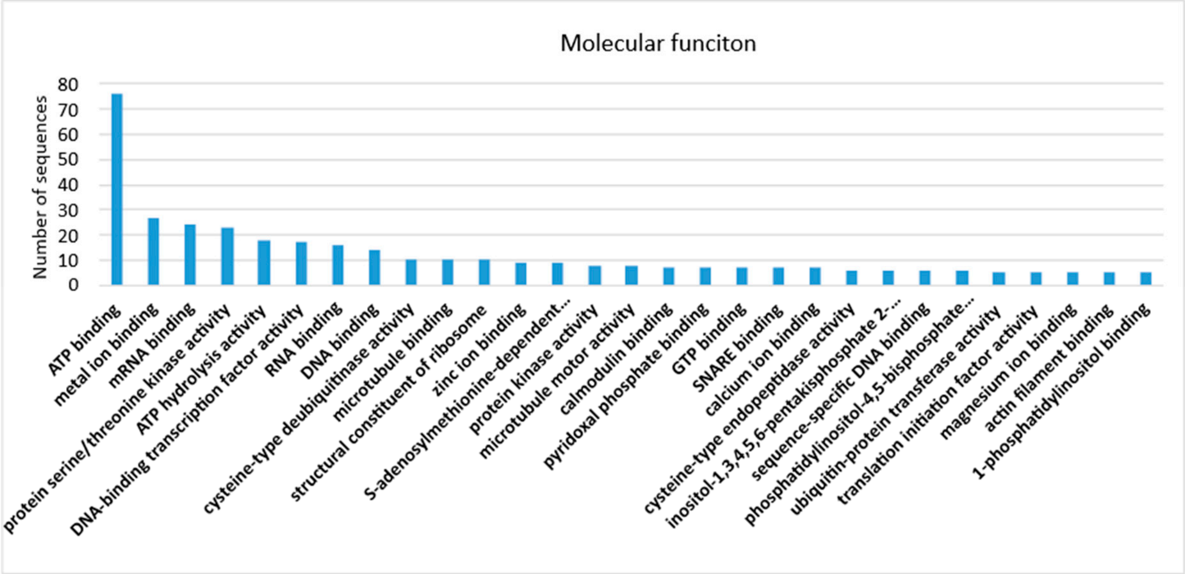

c

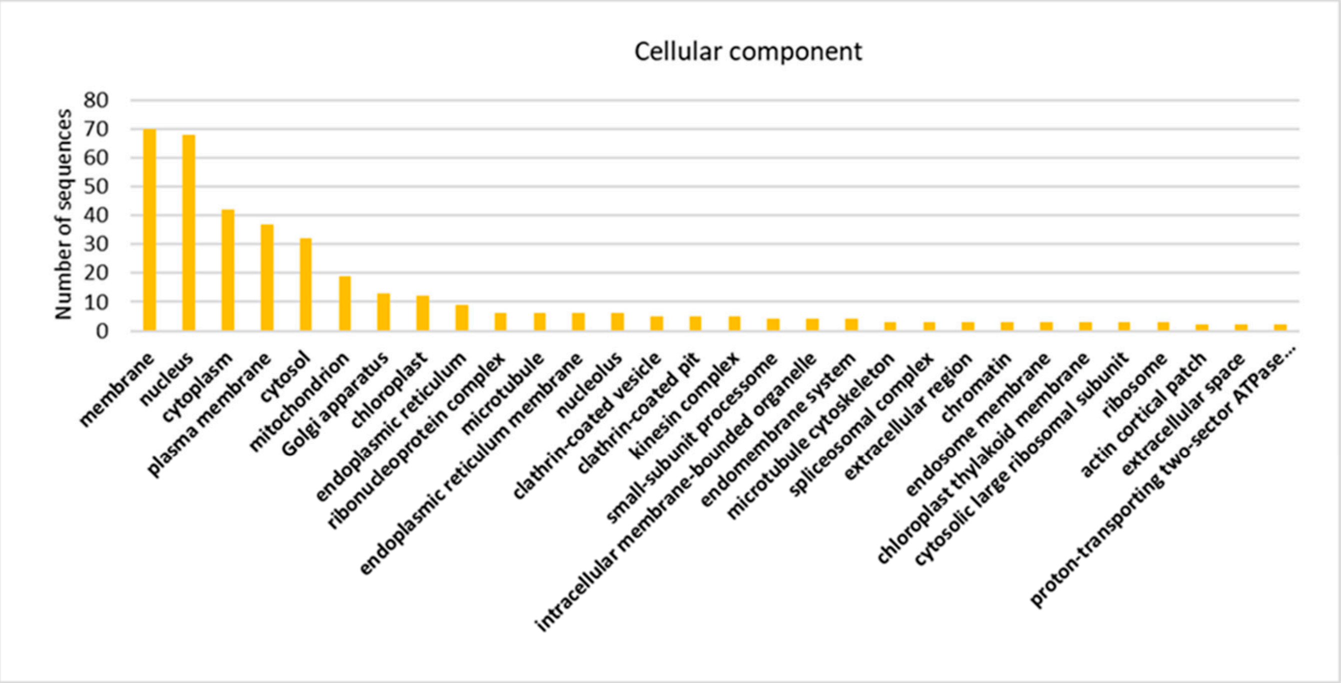

Table S1

FDR values of the phytohormone-related GO terms identified in the GSEA analysis of the investigated sample pairs. This data supplements the Figure 7.

GO term	Hormone	Comparison	Adjusted p-value (FDR)
Salicylic acid mediated signaling pathway	SA	1vs0	1.8×10^{-3}
Response to salicylic acid	SA	1vs0	2.4×10^{-3}
Auxin transport	AUX	2vs0	4.1×10^{-3}
Response to abscisic acid	ABA	2vs0	6.2×10^{-4}
Cellular response to abscisic acid stimulus	ABA	2vs0	2.7×10^{-3}
Abscisic acid-activated signaling pathway	ABA	2vs0	1.3×10^{-2}
Response to jasmonic acid	JA	3vs0	3.6×10^{-4}
Jasmonic acid mediated signaling pathway	JA	3vs0	2.9×10^{-3}
Regulation of JA mediated signaling pathway	JA	3vs0	8.7×10^{-3}
Cellular response to JA stimulus	JA	3vs0	1.1×10^{-2}
Cytokinin-activated signaling pathway	CK	3vs2	3.3×10^{-3}
Response to cytokinin	CK	3vs2	7.6×10^{-3}
Cellular response to cytokinin stimulus	CK	3vs2	1.5×10^{-2}
Ethylene-activated signaling pathway	ET	3vs2	5.8×10^{-3}
Cellular response to ethylene stimulus	ET	3vs2	9.2×10^{-3}
Response to gibberellin	GB	1vs0	4.5×10^{-3}
